# Non-Traditional Risk Factors of Albuminuria in the Pediatric Population: A Scoping Review

**DOI:** 10.3390/ijerph14101231

**Published:** 2017-10-16

**Authors:** Erick Sierra-Diaz, Alfredo de Jesus Celis-de la Rosa, Felipe Lozano-Kasten, Alejandro Bravo-Cuellar, Mariana Garcia-Gutierrez, Hernandez-Flores Georgina

**Affiliations:** 1 Public Health Department, University of Guadalajara, Sierra Mojada 950, Colonia Independencia, Guadalajara, Jalisco 44340, Mexico; alfredo_celis@yahoo.com; 2 Environmental Health Department, University of Guadalajara, Sierra Mojada 950, Colonia Independencia, Guadalajara, Jalisco 44340, Mexico; f_lozano_k@hotmail.com; 3 Immunology Department, Western Research Biomedical Center (IMSS), Sierra Mojada 800, Colonia Independencia, Guadalajara, Jalisco 44340, Mexico; abravoc@prodigy.net.mx (A.B.-C.); gina.geodic1967@gmail.com (H.-F.G.); 4 Pediatrics Department, Hospital Angeles del Carmen, Health Services, Tarascos 3473 Interior 240A, Fraccionamiento Monraz, Guadalajara, Jalisco 44670, Mexico; mariana.endoc@gmail.com

**Keywords:** albuminuria, pediatric population, risk factor

## Abstract

The presence of albumin in urine has been used for more than four decades as a marker of renal and cardiovascular damage. Most of the information on this marker is related to adults. The prevalence of albuminuria in the pediatric population has been reported as being 2.2–12.8% in some countries. Most research in this field is related to albuminuria and diseases, such as diabetes and hypertension. Using the methodology described by Arksey and O’Malley in 2005, a scoping review was carried out to show that the presence of albumin in urine in the pediatric population might be associated with environmental, demographic, congenital, infectious, and non-infectious factors. The information collected is supported by 74 references present in PubMed. The results reveal the multiple causes associated with albuminuria in the pediatric population. This information can be very useful for clinical practice by adding knowledge about albuminuria behavior in children.

## 1. Introduction

At the end of the 1960s, Peterson et al. determined that quantitative analysis of albumin in urine was useful to detect renal alterations [1]. Since then, albumin levels in urine have been used clinically as a tool to measure kidney failure in patients with diabetes mellitus and hypertension [2,3]. Once the relationship between albuminuria and kidney failure was established, quantification methods were developed to be faster and more precise [4].

At present, albuminuria is known as a renal and cardiovascular predictor not only in diabetic patients, but also in those who are apparently healthy. The reported prevalence in general population is 7.2% [5]. The majority of studies have been conducted in adults, despite the interest in early detection having risen recently due to future effects. In Japan, this method has been used since 1979 to detect kidney diseases in the pediatric population [6]. Other countries, such as Australia, the U.K., Norway, the U.S., and Italy, have also carried out this practice since the mid-1980s [7]. Information on prevalence is limited but is reported as approximately 7% [8]. Another study reported that the median ACR (albumin/creatinine ratio) of the random morning urine specimen for children <10 years was 2.91 vs. 2.28 mg/g (*p* < 0.001) for children ≥10 years. The median urinary albumin concentration for children <10 years was significantly higher than in children ≥10 years (2.53 vs. 2.09 μg/mL; *p* = 0.008) [9]. More recently, an Australian study considered 975 children aged 5–18 years. The overall prevalence of albuminuria, as defined by an ACR greater than 30 mg/g, was 12.8% (95% CI 9.9–15.6). In males, the frequency of albuminuria was 10.2% (95% CI 6.1–14.2) and in females, it was 15.5% (95% CI 10.7–20.3) [10].

Orthostatic proteinuria is the most common cause of a positive result of protein (albumin among the various proteins in urine) in pediatric patients (often tall, physically active adolescents). In this circumstance, the detection of isolated proteinuria (in the absence of hematuria) in an asymptomatic individual based on a random specimen during the day must be confirmed by repeating the test (dipstick) on a specimen collected immediately upon patient’s awakening in the morning [11]. In 2010, Brandt et al. characterized the 24-h and diurnal variability of urinary protein excretion and identified the prevalence of orthostatic proteinuria (OP) in healthy children. Upright, supine and 24-h total urinary protein (UrTP) was measured in 91 healthy children aged 6–19 years. The UrTP concentration (mg/dL) was measured using a pyearsocatechol violet method. All participants had normal serum creatinine levels. The mean 24-h UrTP excretion was 64 mg/m^2^ (SD 65).

An elevated 24-h UrTP of >100 mg/m^2^ was found in 18 (19.8%) participants. These 18 participants had orthostatic proteinuria. Three participants (3%) had a normal 24-h protein but an elevated supine protein excretion rate (range of 4.1–5.5 mg/m^2^/h), with a mean UrTP of 108 mg (69 mg/m^2^/24 h) Absolute UrTP excretion (mg/24 h) was higher in older compared with younger children (*p* < 0.05) and was higher in boys than girls (*p* < 0.05). This concluded that children with mild, asymptomatic proteinuria should have a first-morning urine UPcr (urinary protein to creatinine ratio) to rule out OP before further testing and consideration of a referral. Children with normal first-morning urinary protein do not require extensive testing for renal disease and can be monitored yearly for evidence of changing urinary protein excretion [12].

Extensive information already exists in the literature related to the relationship between albuminuria and diseases, such as diabetes, hypertension, cardiopathies, human immunodeficiency virus (HIV) infection, as well as different nephropathies in the pediatric population. The aim of the present study was to review all available information related to the relationship between albuminuria and less common risk factors other than those previously mentioned, as well as to discuss to the association of albuminuria with demographic, pathological, and environmental factors. Finally, the purpose was to alert the reader about the vast diversity of factors causing albuminuria, in order for these to be taken into account when applying detection tests in pediatric population.

## 2. Materials and Methods

The methodology described by Arksey and O’Malley was used [13]. First, a question for the literature review was defined and an information search was subsequently initiated, with PubMed being the main index considered. At the beginning, the key words were albuminuria, children, preschool, and risk factors. Notably, when combining such words, many terms related to articles on diabetes appeared. Thus, we employed the Boolean operator “not” before “diabetes” in order to decrease the number of results. Table 1 graphically depicts the search strategy and results obtained from PubMed.

The main reference selection criteria were: English issues, empirical quantitative and pediatric population research. All articles included were full texts. Papers excluded were those related to: diabetes mellitus, systemic arterial hypertension, HIV infection, any stage of chronic kidney failure, common nephropathies, transplanted patients, oncologic diseases studies and hospitalized patients. In addition, reviews and manuscripts with only an abstract were not considered.

The search, review and selection of papers were performed for eight weeks (September–November 2016) and manuscripts were in the year range of 1981–2016. A total of 74 references were considered (57 for the review and 17, complementary ones). Due to importance of the information, an article published in 2017 was included in the final version of this manuscript.

The selected manuscripts were analytically systematized in an excerpt matrix to organize them easily. For each article, the excerpt matrix considered the following: main author; country of origin; date; design; sample characteristics; data analysis and results. After all articles were processed, a summary of the results was created, which was gathered according to their etiologic factor. Table 2 summarizes the characteristics of the included studies.

## 3. Results

### 3.1. Environmental Factors 

Some children and adolescents are often exposed to environmental factors, which can affect their health in the pediatric stage in addition to having serious consequences later on in the future. Several elements have been reported as being detrimental to people’s health, some of which have been associated with direct kidney damage. Bisphenol A (BPA) is a compound found in products that are consumed daily. Exposure of the pediatric population to this element has been studied for more than a decade [14]. By 2011, canned soup was demonstrated to be related with high BPA levels in urine [15]. Simultaneously, another study demonstrated the possibility of health damage in the pediatric population due to BPA exposure [16]. To date, scientific evidence has demonstrated a relationship between urinary BPA and albuminuria in this population [17]. BPA can co-exist with other pollutants, such as phthalates, which have also been shown to be correlate with consumption levels and can be detrimental to one’s health, specifically causing albuminuria [18,19,20].

Distinct components in the environment (heavy metals) also result in renal toxicity. Lead and cadmium are heavy metals that, together with high- or low-molecular weight proteins, have been associated with albuminuria in pediatric and adult urine [21,22]. Other molecules, as chromium, arsenic and mercury, have been studied to determine levels of damage to the kidney and their relationships with albuminuria [23,24]. However, different authors have not found any relationship between albuminuria and such elements in the pediatric population [25].

Cigarette smoke is among pollutants in the environment that are also related to albuminuria in the early stages. In 2007, a study in adults revealed a relationship between smoking and albuminuria [26]. In 2013, another study in the pediatric population reported independent associations between chronic kidney disease, smoking and proteinuria [27]. At the same time in the U.S., a different group showed a relationship between urinary cotinine and albuminuria in a multi-ethnic study [28]. Therefore, there is an important role of pesticide exposure in these situations, even more so in pediatric working populations. The scientific evidence points to an association, probably cyclic, between pesticide exposure (xenobiotics) and nicotine with albuminuria in vulnerable groups [29].

### 3.2. Non-Transmissible Diseases

The relationship between albuminuria and chronic diseases, such as diabetes mellitus and arterial hypertension, has been previously mentioned [2,3]. However, there are other non-transmissible pathologies related to albuminuria. Sickle-cell anemia is an autosomal recessive disorder associated with chronic kidney disease. Two trials have reported that the prevalence of albuminuria was 15.5–20.7% in separate groups of 90 and 410 pediatric patients, establishing it as an early marker of kidney failure [30,31]. In 2014, a multi-national study in Africa with 2582 patients with sickle cell disease revealed that the prevalence of albuminuria was 29.2% in general and 27% in children younger than 10 years old [32]. The study determined that the frequency of albuminuria varies according to age and country with a higher frequency of 46% in Cameroon (32).

Some other studies consider obesity as a risk factor for albuminuria. In 2014, a study in 901 children aged 6–16 years, 565 of whom were obese, found no difference in albuminuria levels with or without obesity [33]. However, these authors determined that albuminuria was directly related with glomerular filtration rate [33]. Additionally, the prevalence of albuminuria in overweight and obese infants has been shown to be 2.7–4%, which does not differ significantly from estimations in the general population [34,35]. Meanwhile, some other reports indicated that in non-obese adolescents, the prevalence of albuminuria is 8.7% compared to 0.3% in obese adolescents [36].

Respiratory disorders are not excluded as risk factors for albuminuria in the pediatric population. A study conducted in Greece [37], reported an association between albuminuria and severe obstructive sleep apnea in children aged 2–14 years, which occurred as a result of hypoxemia (oxidative stress). Congenital hypothyroidism and Kwashiorkor disease have also been studied, although no relationship with albuminuria has been established to the best of our knowledge [38,39].

### 3.3. Transmissible Diseases

Some infectious diseases have been related to albuminuria, which are mainly transmitted by parasites, bacteria, and viruses. Regarding parasites, having urogenital infections by *Schistosoma haematobium* is considered as a high risk in African countries, especially in the pediatric population. Several studies have reported that this condition is associated with albuminuria and the albumin/creatinine ratio when comparing frequencies between infected and healthy patients [40,41,42]. Furthermore, this has established it as diagnostic marker for the disease and as a way to monitor early complications, particularly in preschool children. Several studies have related intestinal parasites to chronic and acute renal diseases in infants and adults who had no albuminuria, despite exhibiting abnormalities in the general urine test [43,44]. In 2014, a study in India included children older than 11 years of age and adults, with the goal of describing with the relationship of renal disease with *Orientia tsutsugamushi* infections [45]. Results indicated that 55% of infected patients presented with albuminuria. Moreover, another study in 2010 revealed the association between albuminuria and *Leishmania* infection, with a prevalence of 42% in infected patients [46].

Albuminuria in patients with a urinary tract infection (UTI) has also been studied, which found statistically significant differences in pediatric groups with and without UTI in terms of albuminuria levels and albumin/creatinine ratio [47]. Other reports demonstrate an incidence of 51% of albuminuria in pediatric patients with renal scars, secondary to pyelonephritis [48]. The authors of reference [48] utilized this parameter as a long-standing biologic marker. In 1999, a trial in children aged 1.9–16 years demonstrated an association of albuminuria with recurrent tonsillitis (*Streptococcus pyogenes*), recurrent-hypertrophic tonsillitis and hypertrophic tonsillitis with a prevalence of 50%, 38.5%, and 20% respectively, at the time of the tonsillectomy, before there was a decrease six months after surgery [49]. In 2013, an evaluation in Vietnam failed to determine an association of dengue infections and other variables with albuminuria in pediatric patients [50]. The prevalence of albuminuria in both groups was 12%.

### 3.4. Uncommon and Congenital Nephropathies Causing Albuminuria

Hemolytic Uremic Syndrome (HUS) is considered an infectious disease sequalae with potential renal damage. In reports of cases and controls, a frequency of albuminuria of 40% compared to 3.3% albuminuria was found in children with a HUS background and their healthy controls, respectively [51]. Likewise, follow-up studies in patients with this background had results evaluated 3–5 years after the outbreak. Albuminuria frequencies in these patients were 32% and 20%, respectively, compared to healthy subjects [52,53]. Familial Mediterranean Fever (FMF) is an autosomal recessive disease defined by fever episodes, renal polyserositis, and amyloidosis. An experiment with 50 children with FMF and healthy ones measured kidney function in both groups. The authors found a significant difference only in regard to albuminuria and suggested it as a useful monitoring tool in order to decrease future kidney complications [54].

### 3.5. Congenital Urinary Tract Malformations 

A solitary functioning kidney is a clear example of renal mass-decrease function in which hyperfiltration consequences could be expected. This fact could be applied to subjects with congenital or acquired solitary functioning kidneys. A research group in 2006 determined the solitary functioning kidney etiology in a group of patients <18 years of age [55], which was 49% due to congenital renal agenesis (CRA), 41% secondary to nephrectomy and the remainder due to different causes. In patients with congenital renal agenesis, the average follow-up was 9.1 years. The frequency of albuminuria in all the groups was 12.6%, while it was 31.6% in the congenital renal agenesis group. Finally, in patients with a nephrectomy background, a frequency of 7.9% was found. Studies in cases and controls determined in 2014 that there was no significant difference between glomerular filtration and albuminuria in healthy or in subjects with a solitary functioning kidney. The authors also reported that changes in kidney function and albuminuria are directly related with time and patients with a solitary functioning kidney had a higher hyperperfusion risk [56]. Another study tracked a group of 42 children with a solitary functioning kidney for 11.3 years [57]. The frequency of albuminuria in 42 of these children was 7.1%, while this frequency was 7.4% and 6.4% for the groups with congenital renal agenesis and a solitary functioning kidney, respectively.

Autosomal dominant polycystic kidney disease (ADPKD) is one of the most frequent inheritable conditions. In 1998, a report in 189 children of families with this condition revealed that of the 103 of them with ADPKD-positive results, 72% had albuminuria, while in the non-affected group, a frequency of 42% was found [58]. In the same year, there was a follow-up study in subjects aged 4–21 years with ADPKD and normal kidney function, in which patients were divided into three groups according to hypertension and ADPKD severity. This study reported an average of 25.3 mcg/day of urinary albumin in the three groups, which did not significantly differ [59]. Later, albuminuria was used as an early renal failure marker in children aged 1–17 years as there was a frequency of 58% for albuminuria in the study group [60].

Other congenital entities, such as vesicoureteric reflux (VUR), have also been studied, revealing a direct proportional relationship between albuminuria levels and the degree of nephropathy secondary to VUR [61]. A study in 2007 reviewed retrospectively the notes of 176 children born in 1970–1998 that were diagnosed with chronic renal failure (CRF). Seventy-four (42%) children had renal failure secondary to urinary tract obstruction and 102 children (58%) secondary to renal dysplasia alone (*n* = 41) or with VUR (*n* = 61). There was a significant relationship between the amount of albuminuria and the rate of deterioration (*p* < 0.001). Children with ACR <50 mg/mmoL showed a median deterioration of −1.5 mL/min per 1.73 m^2^, whereas children with pronounced proteinuria and ACR of >200 mg/mmoL had a median deterioration of –6.5 mL/min per 1.73 m^2^ per year [62].

### 3.6. Demographic Factors

There is evidence related to demographical factors concerning the prevalence of albuminuria according to gender and age [53,63,64,65]. Other scientific research considers that even when there are differences regarding gender, age is inversely proportional to albuminuria levels in boys and girls [66]. Likewise, there are reports on the prevalence of albuminuria (8.3% vs. 2.1%) in newborn infants with low birth weights when compared to normal-weight newborns [67]. Some studies also report a similar albuminuria prevalence in ethnic groups [66], even when others show differences in albuminuria levels in Australian aboriginal and non-aboriginal people [68]. It is important to emphasize that in the latter study, the difference observed in Australian aboriginal people is associated with obesity, which was already discussed previously. In addition, other studies in Australia also present a prevalence of 7.3% for albuminuria in Australian aboriginal people without any difference in non-aboriginal people. Moreover, these authors indicate that neither place-of-residence, age, gender, nor social conditions are factors affecting the prevalence of albuminuria [69].

## 4. Discussion

Albuminuria is defined as the presence of albumin in urine in a range of 30–300 μg/mg [70]. When the urinary albumin level is divided by the urinary creatinine value, the albumin/creatinine ratio is obtained, which is an estimation reported in several studies [71]. Thus, by employing both parameters, urinary albumin levels and their frequency can be reported in specific populations. The prevalence and variability of albuminuria have already been mentioned as being related to certain pathologies, such as age, gender, etc. However, it is noteworthy that the determination of urinary albumin also depends on instruments from simple test strips to more modern, sensitive, and easy-to-use methods, particularly in children [8,72]. Additionally, the human factor may also be considered as strong determinant in results.

The use of this assay is well established as a screening test in Asian countries, such as Japan, Taiwan, and South Korea. However, this is still global controversy with respect to its use and there is no international consensus, at least in Western countries, related to the benefit of its use in children or adolescents [73]. Some other authors consider the use of test strips as an economic, but not-very-helpful, screening tool [74].

In general, and based on the present study, the use of this marker might present several advantages in cardiovascular and early renal conditions. Nevertheless, this is mainly for the populations at risk that are already defined, as it is not clear whether their detection in relatively healthy patients is appropriate when there is a lack of risk factors. Furthermore, among the environmental factors mentioned, including BPA, phthalates and heavy metals, more long-term studies are needed to define their real importance as risk factors. In addition, from this review, the cyclic nature of albuminuria is evident, especially in children. Exposure (even temporary) to risk factors should also be taken into account when testing. Moreover, in terms of the variable nature of this marker, its inversely proportional relationship with age must be added. All these details are relevant when considering that prevalence in chronic kidney disease, with non-diabetic and non-hypertensive patients, does not match with the prevalence of albuminuria in pediatric populations without renal disease.

## 5. Conclusions

Finally, it is possible to conclude that urinary albumin detection is a useful tool with prognostic value. However, there are some gaps regarding its use in general pediatric population and its role in several diseases is not clear. Changes in the prevalence of albuminuria within a specific place and in the pediatric population could be explained by taking in account several associated factors due to its probable cyclic behavior. Achieving reliable results would depend on diagnostic tests, particular cases and only in massive case detection, controlling, and considering all possibilities affecting the nature of urinary albumin.

## Figures and Tables

**Table 1:** Keyword combination and PubMed results.

	Key-Words Combination	Number of Hints	Final Selection
PubMed	Albuminuria, risk factor and children	298	59
PubMed	Albuminuria, risk factor and non-diabetic children	87	12
PubMed	Albuminuria and non-diabetic preschoolers	291	31

**Table 2:** Characteristics of the studies that only included subjects under 21 years old.

Author (Year) Country [ref]	Study Design	Sample (Age)	Sample and Method (Albuminuria)
Trasande (2013) USA [17]	Cross-sectional analyses	N = 710 (6–19 years)	First morning urine sample. Solid-phase fluorescent immunoassay
Tsai (2016) China [19]	Cross-sectional analyses	N = 184 (<10 years)	Spot urine sample. Radioimmunoassay (RIA) using albumin RIA kit
Trasande (2014) USA [20]	Cross-sectional	N = 667 (6–19 years)	First morning urine sample. Solid-phase fluorescent immunoassay
Chan (2012) China [21]	Cross-sectional	N = 3102 (1–21 years)	Spot urine sample. Immunoturbidimetric assay
Noonan (2002) USA [22]	Case-control	N = 159 (6–17 years)	Spot urine sample. Enzyme immunosorbent assay
Cardenas (2016) Mexico [23]	Cross-sectional sampling	N = 107 (5–12 years)	Spot urine sample. Turbidimetry method on Radox Daytona
Kong (2012) China [25]	Cohort	N = 120 (12–19 years)	Morning urine sample. Immunoturbidimetric assay
Omologa (2013) USA [27]	Cohort	N = 366 (1–16 years)	Spot urine sample. Method not described
Nascimento (2017) Brazil [29]	Cohort	N = 66 (6–12 years)	Two urine samples 6 months apart. Immunoturbidimetric assay
Becton (2010) USA [30]	Cross-sectional	N = 90 (2–18 years)	Two urine samples 6 months apart. Immunoturbidimetric assay
McPherson (2011) USA [31]	Cross-sectional	N = 410 (2–21 years)	Spot urine sample. Radioimmunoassay (RIA)
Ranque (2014); five countries in sub-Saharan Africa [32]	Cross-sectional	N = 2582 (N = 527 <10 years)	Spot urine sample. HemoCue Albumin 20 system or Siemens Clinitek StatusAnalyzer
Di Bonito (2014) Italy [33]	Cross-sectional	N = 901 (6–16 years)	First morning urine sample. Kinetic nephelometric method.
Lurbe (2013) Spain [34]	Cross-sectional	N = 134 (9–18 years)	First morning urine sample. Immunonephelometric assay
Radhakishun (2013) The Netherlands [35]	Cross-sectional	N = 408 (3–19 years)	Morning urine sample. Immunochemistry system
Nguyen (2013) USA [36]	Cross-sectional	N = 2515 (12–19 years)	Morning urine sample. Solid-phase fluorescent immunoassay
Varlami (2013) Greece [37]	Case-control	N = 129 (2–14 years)	Two urine samples: 10 p.m. and 8 a.m. h. Immunonephelometric assay
Wami (2015) Zimbabwe [40]	Cross-sectional	N = 298 (1–10 years)	Morning urine sample. Clinitek Microalbumin Reagent Strips
Stothard (2008) Zanzibar [41]	Cross-sectional	N = 66 (9–15 years)	Mid-morning urine sample. Hemastix_reagent strips and Microalbustix_ reagent strips
Sousa-Figueiredo (2009) Unguja, Tanzania [42]	Cross-sectional	N = 140 (9–15 years)	Mid-morning urine sample. Albumin-HemoCue photometer
Elnojomi (2010) Sudan [46]	Cross-sectional	N = 88 (children age not available)	24-h urine sample. Turbidimetric kit and ELISA
Karlen (1996) Sweden [48]	Cross-sectional	N = 57 (1.7–17.9 years)	Four urine collection periods lasting 30 min each were obtained in every subject. Solid-phase radioimmunological assay
Lopez-Gonzalez (1999) Spain [49]	Cohort	N = 90 (1.9–16 years)	Two 24-h urine samples 2 months apart. Laser nephelometry
Hanh Thien (2013) Vietnam [50]	Prospective descriptive study	N = 429 (5–15 years)	Spot urine sample. ELISA using rabbit anti-human albumin polyclonal antibodies and a human serum albumin standard
Sharma (2010) Canada [51]	Cohort	N = 48 (3–18 years)	Spot urine samples. Immunoassay
Garg (2005) Canada [52]	Cohort	N = 19 cases (1–5 years) N = 38 controls	Spot urine sample. Solid-phase competitive chemiluminescent enzyme immunoassay
Garg (2008) Canada [53]	Cohort	N = 19 cases (4–8 years) N = 64 controls	Two-first morning urine samples. Image Beckman Coulter immunoassay
Ergüven (2008) Turkey [54]	Cross-sectional	N = 50 cases (3–19 years) N=20 controls (3–17 years)	24-h urine sample. Immunoturbidimetric assay
De Lucas (2006) Spain [55]	Prospective	N = 95 (1–17 years)	Spot urine samples. Method not described
Shirzai (2014) Turkey [56]	Cross-sectional	N = 44 cases (6–16 years) N = 25 controls (5–10 years)	24-h urine sample. Nephelometry
Kolvek (2014) Slovakia [57]	Prospective follow-up study	N = 42 (mean 11.3 years)	24-h urine sample. Method not described
Sharp (1998) USA [58]	Cross-sectional	N = 103 cases (mean 11.2 years) N = 86 controls (mean 10.6 years)	Spot urine sample. Radioimmunoassay (RIA)
Cadnapaphornchai (2009) USA [59]	Clinical Trial	N = 85 (4–21 years)	Two 24-h urine sample. Method not described
Selistre (2012) France [60]	Cross-sectional	N = 52 (1–17 years)	Second-morning urine sample. Nephelometry, BM2
Lama (2003) Italy [61]	Retrospective	N = 100 (mean 11.5 years)	Two 24-h urine samples. Enzyme immunoassay
Gonzalez (2007) England [62]	Retrospective	N = 176 (0–11.9 years)	Spot urine sample (MNS). Method not described
Davies (1984) England [63]	Cross-sectional	N = 400 (4–16 years)	24-h urine sample. ELISA
Gracchi (2015) The Netherlands [64]	Cohort	N = 1352 (24–60 months)	Pantyliners. Nephelometry
Wu (2014) China [65]	Cross-sectional	N = 1986 (6–19 years)	Morning spot urine sample. Immunoturbidimetric assay
Trachtenberg (2007) USA [66]	Secondary analysis of a clinical trial	N = 534 (6–10 years)	Two urine samples 2 years apart. Nephelometric immunochemical methods
Kim (2017) Australia [68]	Cohort	N = 3418	Spot urine sample. Dipstick analysis Siemens Clinitek machine
Haysom (2007) Australia [69]	Cross-sectional	N = 2266 (4–14.8 years)	Morning urine sample. Dipsticks Clinitek 50 machine
